# Effective intraperitoneal gene transfection system using nanobubbles and ultrasound irradiation

**DOI:** 10.1080/10717544.2017.1319433

**Published:** 2017-04-27

**Authors:** Koyo Nishimura, Shintaro Fumoto, Yuki Fuchigami, Masayori Hagimori, Kazuo Maruyama, Shigeru Kawakami

**Affiliations:** 1 Graduate School of Biomedical Sciences, Nagasaki University, Nagasaki, Japan and; 2 Faculty of Pharma-Sciences, Teikyo University, Tokyo, Japan

**Keywords:** Bubble liposomes, ultrasound, plasmid DNA, intraperitoneal injection, gene therapy

## Abstract

In this study, we demonstrate the low toxicity and highly efficient and spatially improved transfection of plasmid DNA (pDNA) with liposomal nanobubbles (bubble liposomes [BLs]) using ultrasound (US) irradiation in mice. Naked pDNA with BLs was intraperitoneally injected, followed by US irradiation. The injection volume, the duration of US irradiation, and the dose of BLs were optimized. Both BLs and US irradiation were essential to achieve high transgene expression from naked pDNA. We observed transgene expression in the entire peritoneal tissues, including the peritoneal wall, liver, spleen, stomach and small and large intestines. The area of transfection could be controlled with focused US irradiation. There were few changes in the morphology of the peritoneum, the peritoneal function or serum alanine aminotransferase levels, suggesting the safety of BLs with US irradiation. Using a tissue-clearing method, the spatial distribution of transgene expression was evaluated. BLs with US irradiation delivered pDNA to the submesothelial layer in the peritoneal wall, whereas transgene expression was restricted to the surface layer in the liver and stomach. Therefore, BLs with US irradiation could be an effective and safe method of gene transfection to the peritoneum.

## Introduction

Development of intraperitoneal gene transfection system is required for the therapy of peritoneal diseases (e.g. peritoneal fibrosis). Intraperitoneal gene transfection using viral vectors, such as recombinant adenoviral vectors, has been shown to achieve highly effective transfection in peritoneal tissues (Mahasreshti et al., 2001). However, viral vectors incur several problems, including the host immune responses. Moreover, the intraperitoneal readministration of viral vectors is difficult because neutralizing antibodies are produced against the vectors (Hoff, 2001). In contrast, nonviral transfection using naked plasmid DNA (pDNA) is a simple method with high safety. However, the transgene expression using naked pDNA is generally insufficient, because it is rapidly degraded by nucleases (Kawabata et al., 1995). The hydrodynamic injection of naked pDNA, a highly effective transfection method (Liu et al., 1999), is not applicable to intraperitoneal gene transfection because the peritoneal cavity has a capacity to cancel the high pressure of a hydrodynamic injection. Therefore, gene carriers such as cationic liposomes (Lee et al., 2002) are generally required to improve nonviral gene transfer. However, there are concerns about the cytotoxicity and low tissue selectivity of cationic carriers, and so, development of a safe and tissue-selective intraperitoneal gene transfection method is important.

A transfection method using microbubbles with ultrasound (US) contrast gas, that is, sonoporation, is a cell- or tissue-selective gene transfection method with reportedly low cytotoxicity (Liu et al., 2006). A sonoporation method using microbubbles and US irradiation achieved effective intraperitoneal gene transfer (Guo et al., 2007), but transgene expression was only detected in limited peritoneal tissues, such as the peritoneal wall and adipose tissues, and lasted for only 2 weeks. To develop a gene therapy method for peritoneal diseases, high transfection efficacy, long-term gene expression and a high level of safety are essential. The pharmaceutical stability of micrometer-sized particles is generally low, so US-responsive nanometer-sized particles are preferable for future clinical use.

In previous studies, polyethylene glycol (PEG)–liposomes with a US contrast gas, called “liposomal nanobubbles” (bubble liposomes; BLs), have been developed as nanosized gene transfection agents (Suzuki et al., 2008). Kodama et al. showed that perfluoropropane gas was trapped within the BLs (Kodama et al., 2010). Gene transfection by BLs with US irradiation is expected to be a useful method because BLs are more pharmaceutically stable than microbubbles due to the smaller particle size with PEGylation. BLs are also easily modified with targeting ligands in addition to PEGylation. Gene delivery systems using BLs with US irradiation enhance the gene transfection efficiency to targeted sites, such as the liver, kidney and tumors (Un et al., 2010; Kurosaki et al., 2014; Suzuki et al., 2015). Under optimal condition of US intensity and irradiation time, highly efficacious, long-term transgene expression has been achieved (Kurosaki et al., 2014). However, no study has investigated intraperitoneal gene transfection using BLs with US irradiation.

In the present study, we demonstrated the effective and safe intraperitoneal gene transfection using BLs with US irradiation in mice. We optimized the system for high transgene expression. Taking the mechanism of peritoneal fibrosis generation into consideration, it is important to control depth of transgene expression because both mesothelial cells and fibroblasts plays a crucial role in the generation of peritoneal fibrosis (Aroeira et al., 2007). Therefore, we used a previously reported tissue-clearing method (Kuwajima et al., 2013; Fumoto et al., 2016) to evaluate the depth of transgene expression in intraperitoneal tissues when BLs with US irradiation were used.

## Experimental section

### Materials

Reagents and distributers are listed as follows. Paraformaldehyde, formamide, hematoxylin and eosin: Wako Pure Chemical Industries Ltd. (Osaka, Japan). Triton X-100: Nacalai Tesque (Kyoto, Japan). Ethylenediaminetetraacetic acid disodium salt (EDTA-2Na): Dojindo Laboratories (Kumamoto, Japan). Tetramethylrhodamine-dextran-10 (RD-10) (MW 10 000): Invitrogen (Carlsbad, CA). Fluorescein isothiocyanate-dextran-2000 (FD-2000; MW 2 000 000) and 1,1′-Dioctadecyl-3,3,3′,3′-tetramethylindocarbocyanine perchlorate (DiI): Sigma-Aldrich Co. (St. Louis, MO). Methoxypolyethyleneglycol 2000-distearoylphosphatidylethanolamine (PEG-DSPE): NOF Co. (Tokyo, Japan). Distearoylphosphatidylcholine (DSPC): Avanti Polar Lipids Inc. (Alabaster, AL).

### pDNA

The pCMV–luciferase vector with a firefly luciferase gene was used and purified as reported previously (Kawakami et al., 2002). The pCpGfree–Lucia vector with a secretable synthetic luciferase Lucia gene was purchased from Invivogen (San Diego, CA). The pZsGreen1-N1 vector with a ZsGreen1 green fluorescent protein gene was purchased from Clontech (Takara Bio Inc., Shiga, Japan).

### Preparation of BLs

DSPC and PEG-DSPE were dissolved in chloroform (94:6 in a molar ratio) and dried by evaporation and subsequently vacuum-desiccated. Then, the lipid film was dispersed in phosphate-buffered saline at 65 °C to produce liposome. The liposome was sonicated for 3 min using a tip sonicator. After that, the liposome was sterilized with a 0.45-μm filter. BLs (2 mL of 1 mg/mL dispersion) were prepared from the liposome as described previously (Suzuki et al., 2007). As the condition of the BLs production, the liposome enclosed with a perfluoropropane gas was sonicated in a bath-type sonicator 1510 J-DTH (42 kHz, 90 W, 5 min; Branson Ultrasonics, Tokyo, Japan). The size and zeta-potential were measured using Zetasizer Nano ZS (Malvern Instruments, Worcestershire, UK). To check the stability of BLs, the turbidity was measured with or without US irradiation. Changes in optical density (OD) at 600 nm of BLs with or without US irradiation were monitored using an ultraviolet and visible spectrophotometer (UVmini-1240, Shimadzu Corp., Kyoto, Japan).

### Animals

We purchased 5-week-old male ddY mice (26–32 g) from Kyudo, Co, Ltd (Saga, Japan). The mice can freely access to a standard laboratory diet (MF, Oriental Yeast Co., Ltd., Tokyo, Japan) and water before experiments. We observed the Guidelines for Animal Experimentation of Nagasaki University. For anesthesia, we used sodium pentobarbital (40-60 mg/kg).

### *In vivo* gene transfer to peritoneal tissues

Mixtures of 60 μg of pDNA (pCMV–luciferase, pZsGreen1-N1, or pCpGfree–Lucia) in saline and 250 μg of BLs or Sonazoid® (Daiichi Sankyo, Co., Ltd., Tokyo, Japan) were injected intraperitoneally into the mice. Immediately after injection, the abdominal area was irradiated transdermally with US (frequency, 1.045 MHz; duty cycle, 50%; burst rate, 10 Hz; intensity, 1.0 W/cm^2^) using a sonicator (Sonopore-4000, Nepa Gene, Chiba, Japan) with a probe (diameter, 20 mm).

### Luciferase assay

To detect the firefly luciferase activity, we performed luciferase assay as described previously (Fumoto et al., 2016). To detect the Lucia luciferase activity, the sample was mixed with 50 μL of the luciferase assay substrate (Quanti-Luc, Invivogen, San Diego, CA). Luciferase activities are presented in relative light units (RLU) per g of tissue or per mL of fluid.

### Histological assessment

For a histological analysis, hematoxylin and eosin (H&E) staining was utilized as described (Hirata et al., 2014). The stained samples were observed with a microscope Axio Vert.A1 (10 × objective lens) equipped with a digital camera AxioCam MRc (Carl Zeiss Microimaging GmbH, Jena, Germany). Acquisition software was ZEN2012 light edition.

### Evaluation of peritoneal function using dual macromolecular markers

Peritoneal function was evaluated using dual macromolecular markers, as previously described (Hirata et al., 2014).

### Evaluation of hepatic toxicity

To assess the hepatic toxicity of BLs with US irradiation, alanine aminotransferase (ALT) activity was determined with the Transaminase CII-Test Wako kit (Wako).

### Observation of the depth of transgene expression

One hour before experiments, 300 μL of 100 μM DiI solution was injected intraperitoneally into the mice to stain the surface cells of the peritoneum. Tissue clearing was performed with the Clear^T2^ protocol, as reported previously (Kuwajima et al., 2013). The cleared organs were observed with an inverted confocal microscope (LSM 710; Carl Zeiss Microimaging GmbH). The laser line used was 488 and 543 nm. The acquisition software was ZEN2012. The objective lens was a 25 × oil-immersion lens LD LCI Plan-Apochromat (working distance 0.57 mm; numerical aperture 0.8).

### Statistical analysis

Statistical comparisons between two groups were made with Student’s *t*-test. For multiple groups, Tukey’s test was performed. A *p* value less than 0.05 was deemed statistically significant.

## Results

### Physicochemical property of BLs

Sonication of liposomes for 5 min in a bath-type sonicator resulted in the disappearance of the original peak and increase in size, indicating successful formation of BLs (Figure S1). The average particle size and zeta-potential of BLs were 314.3 ± 8.392 nm and −1.72 ± 0.569 mV, respectively. To check the stability of BLs, we monitored the turbidity of BLs with or without US irradiation (Figure S2). The OD 600 nm of BLs without US irradiation was unchanged for 3 min. In contrast, the OD of BLs was decreased to one-tenth within 30 s by US irradiation.

### Optimization of intraperitoneal gene transfection conditions

We compared BLs with microbubbles (Sonazoid®) for intraperitoneal gene transfection (Figure S3). BLs tended to increase the transgene expression in several intraperitoneal tissues such as the liver compared with microbubbles, although the changes were not statistically significant.

**Figure 1.**
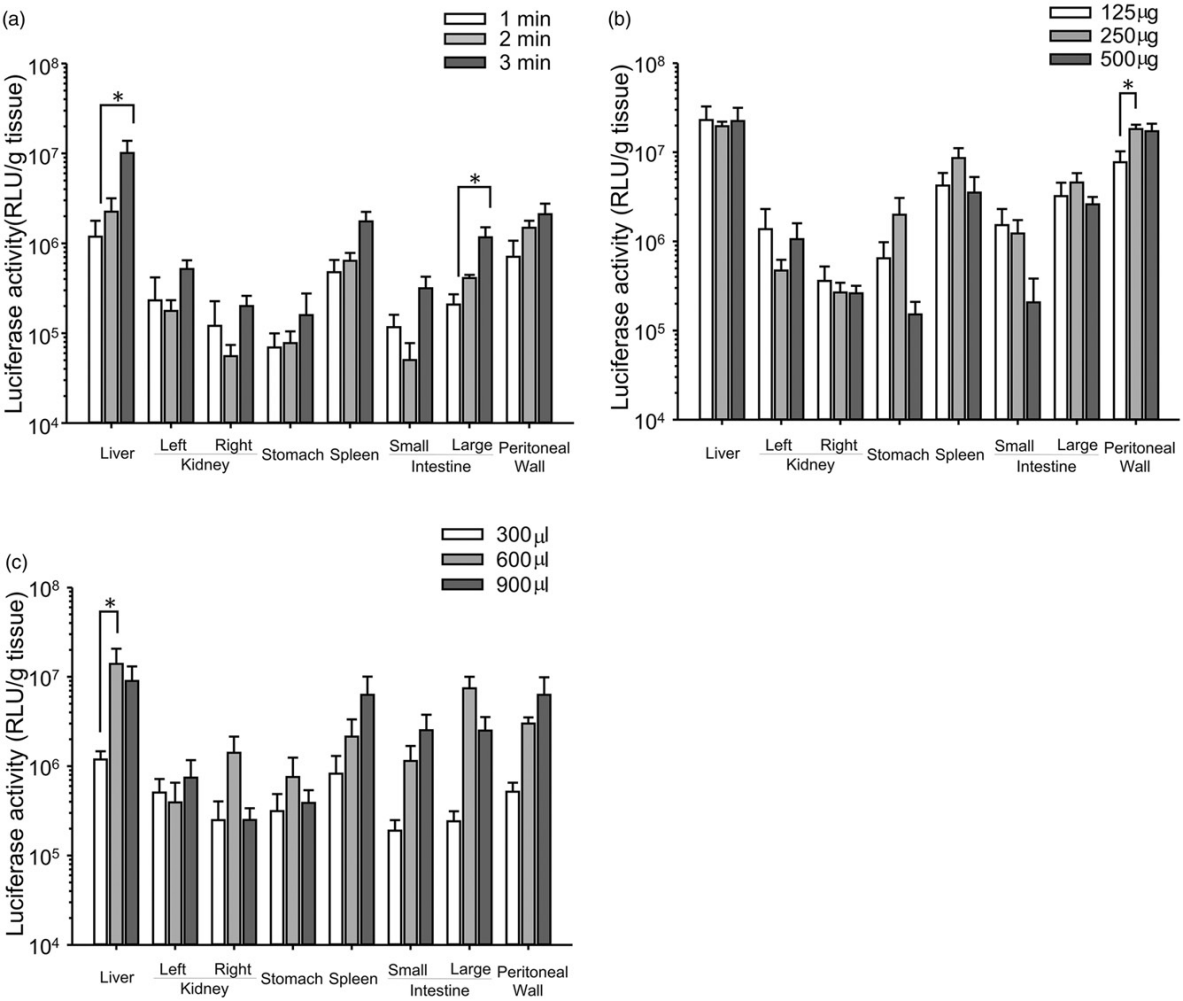
Optimization of the transfection conditions. (a) Effects of the duration of US irradiation, (b) dose of BLs, and (c) injection volume. (a) Injection of a 600 μL mixture of pDNA and BLs (250 μg), followed by US irradiation of different durations. (b) Injection of a 600 μL mixture of pDNA and different doses of BL, followed by US irradiation for 3 min. (c) Injection of several volumes of the mixture (BLs; 250 μg), followed by US irradiation for 3 min. Six hours after injection of pCMV–Luciferase, the luciferase activity was measured. Each bar represents the mean ± SE of four experiments. *p < 0.05.

To obtain high transfection efficiency in peritoneal tissues, the duration of US irradiation, the injection volume and dose of BLs were optimized (Figure 1). The transgene expression achieved in the liver and large intestine with BLs and US irradiation increased as the irradiation time increased (Figure 1a). In the other tissues, transgene expression tended to increase as the duration of US irradiation increased. In contrast, transgene expression did not increase when the dose of BLs was increased (Figure 1b). Significant increases in the liver were observed with an injection volume of 600 μL (Figure 1c) and in the peritoneal wall with an injection dose of 250 μg of BLs (Figure 1b). Therefore, in subsequent experiments, the mice were injected intraperitoneally with a 600 μL mixture of pDNA and BLs (250 μg), followed by US irradiation for 3 min.

We first examined the transgene expression after the peritoneal tissues were treated with BLs only or US irradiation only. We found that the transgene expression achieved with both these methods was less than 2 × 10^5^ RLU/g tissue (data not shown). We then investigated the effective transgene expression in peritoneal tissues when a combination of BLs and US irradiation was used. As shown in Figure 1(a–c), transgene expression after the injection of BLs with US irradiation was 5 × 10^5^–2 × 10^7^ RLU/g tissue, and was altered by changing the transfection conditions, such as the duration of US irradiation (a), the injection volume (b) and the dose of BLs (c).

### Effect of the US irradiation area on transgene expression in each tissue

**Figure 2.**
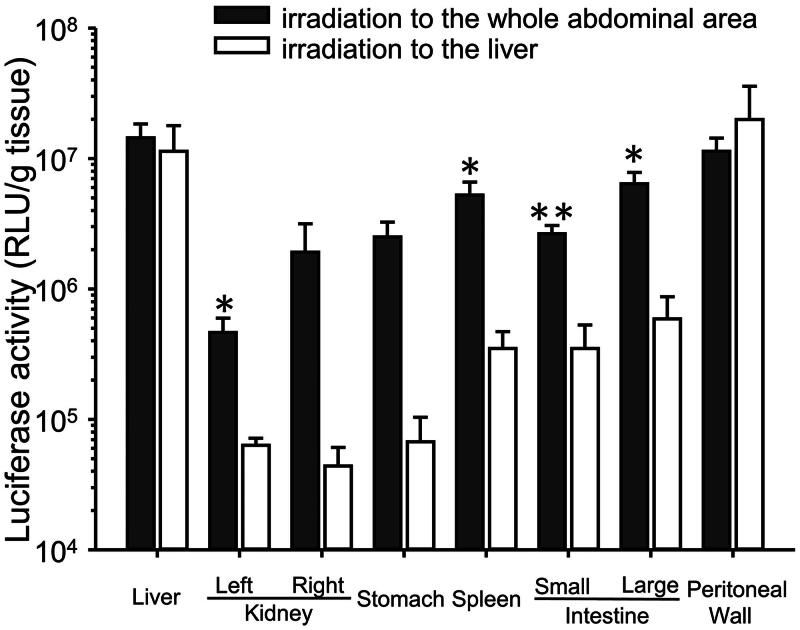
Effect of the area of US irradiation on transgene expression in each tissue. Mice were injected intraperitoneally with a 600 μL mixture of pDNA (60 μg) and BLs (250 μg), followed by US irradiation for 3 min to the liver. Six hours after injection of pCMV–Luciferase, the luciferase activity was measured. Each bar represents the mean ± SE of four experiments.

When the liver was irradiated transdermally with US, transgene expression in the liver and peritoneal wall was higher than that in the non-US-irradiated tissues (Figure 2). The transgene expression in the non-US-irradiated tissues was significantly lower than when the whole abdominal area was transdermally irradiated with US.

### Duration of transgene expression

**Figure 3.**
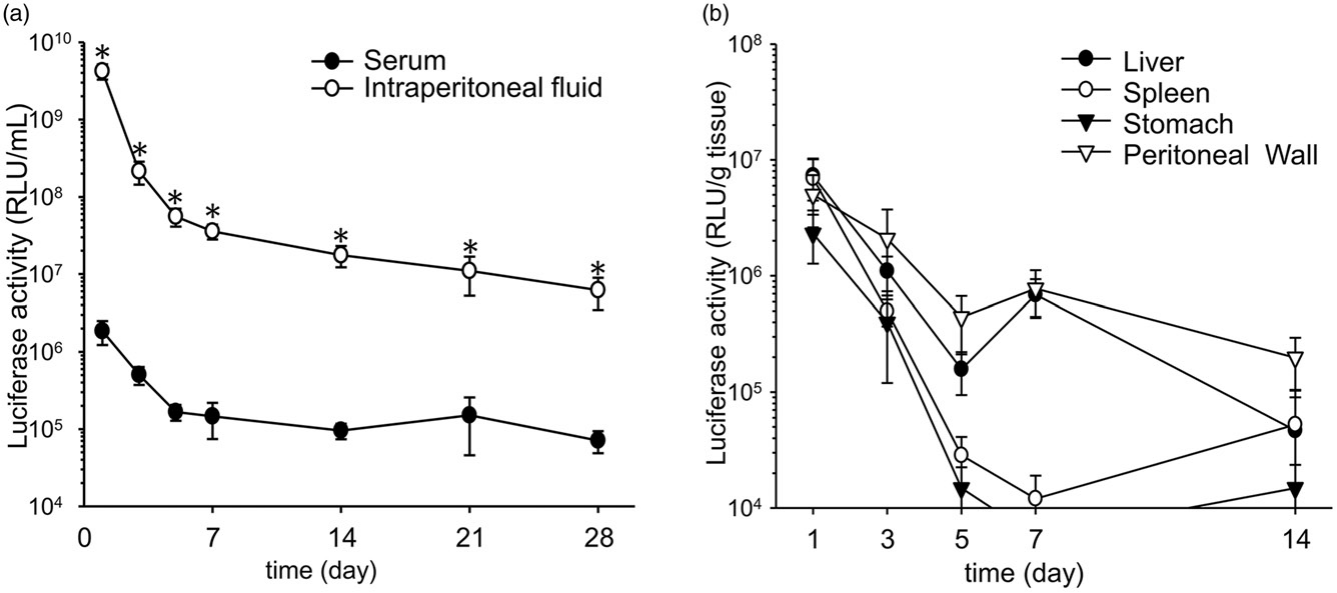
Duration of transgene expression after transfection. The time course was analyzed using pCpGfree–Lucia (a) and pCMV–luciferase (b). Each value represents the mean ± SE of at least four experiments. *p < 0.05 compared with serum.

The time course of transgene expression was monitored. Transgene expression in the intraperitoneal fluid transfected with the CpG-free pDNA vector encoding a secretable form of luciferase persisted and was significantly higher than that in the serum for at least 1 month (Figure 3). The area under the curve for transgene expression in the intraperitoneal fluid calculated with the simplified trapezoidal rule (5.2 × 10^9^ RLU·day/mL) was 900-fold higher than that in the serum (5.8 × 10^6^ RLU·day/mL) (Figure 3a). The transgene expression of pCMV–luciferase in the peritoneal tissues, such as the liver, stomach and spleen, decreased with time (Figure 3b). Transgene expression in the peritoneal wall was detected for at least 2 weeks.

### Assessment of potential toxicity of BLs with US irradiation

**Figure 4.**
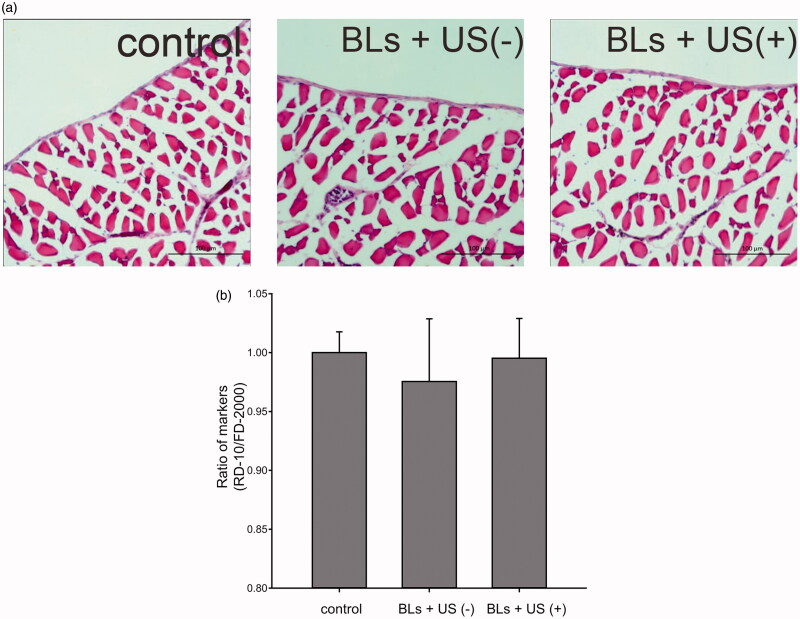
Assesment of peritoneal toxicity after transfection with BLs and US irradiation. Histological assessment with H&E staining of the peritoneal wall (a). Scale bar, 100 μm. Evaluation of peritoneal function by dual macromolecular markers (b). Each bar represents the mean ± SE of four experiments. There were no significant differences.

**Figure 5.**
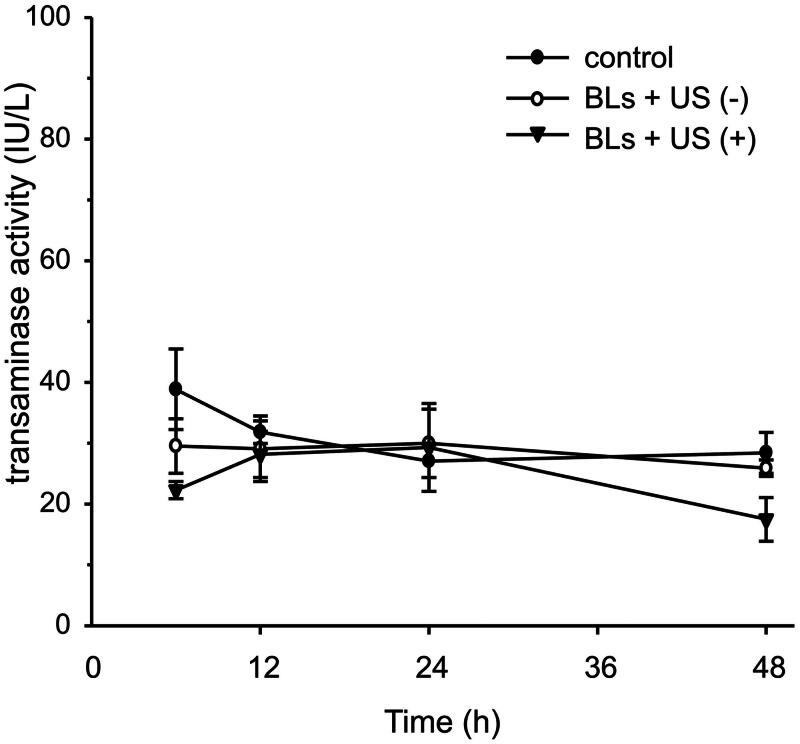
Assessment of hepatic toxicity. Serum ALT activities in the control group, BLs without US irradiation group, and BLs with US irradiation group were determined. Each value represents the mean ± SE of four experiments. The differences were not significant.

To evaluate the potential peritoneal injury caused by transfection, the peritoneal wall was stained with H&E after transfection with BLs and US irradiation (Figure 4a). No histological abnormalities in the peritoneum were caused by BLs with US irradiation. Potential peritoneal injury was also evaluated using dual macromolecular markers, as previously reported (Hirata et al., 2014). The RD-10/FD-2000 ratio, an index of peritoneal function, did not differ between the mice transfected using BLs with US irradiation and the mice injected with saline (Figure 4b). To evaluate hepatic toxicity, serum ALT activity was also monitored. The serum ALT levels in the mice after transfection with BLs and US irradiation did not increase for 48 h, as was also observed in the group treated with BLs but without US irradiation (Figure 5).

### Evaluation of depth of transgene expression

**Figure 6.**
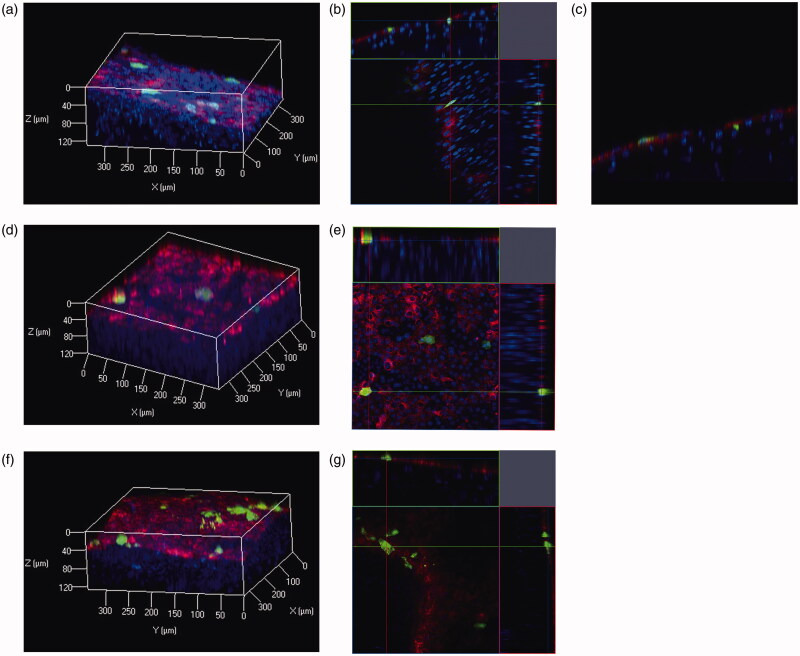
Spatial distributions of transgene expression in peritoneal tissues after pZsGreen1-N1 injection with BLs and US irradiation. Blue signals indicate nuclei, green signals indicate expression of the ZsGreen1 transgene, and red signals indicate DiI-labeled surface cells of the peritoneum. Transgene expression in the peritoneal wall is indicated in (a) three-dimensional (3 D), (b) orthogonal, (c) X–Z plane images. Transgene expression in the liver and stomach is indicated as 3 D (d,f) and orthogonal (e.g.) images (Color version of this figure is available Online).

To assess depth of transgene expression in the peritoneum, we used the Clear^T2^ tissue-clearing method. The surface cells of the peritoneum were labeled with DiI. In the visceral peritoneum, such as the liver and stomach, transgene expression was only observed on the tissue surface (Figure 6d–g). In contrast, transgene expression in the peritoneal wall was detected not only on the tissue surface but also in the submesothelial layer (Figure 6a–c). Thus, BLs with US irradiation showed a slightly improved spatial distribution of transgene expression in the peritoneal wall.

## Discussion

In this study, we successfully optimized the transfection conditions for BLs with US irradiation to enhance the expression of the transgene in various peritoneal tissues, including the liver and peritoneal wall. Among alternative nonviral intraperitoneal gene transfection systems, methods based on cationic liposomes and polymers are cytotoxic and the cationic charge induces an inflammatory response (Wei et al., 2015). We have previously reported that calcium carbonate microflowers can achieve effective and safe intraperitoneal gene transfection (Fumoto et al., 2012), but the transgene-positive tissues or cells in the peritoneal cavity cannot be controlled. The development of a tissue- or cell-specific intraperitoneal gene transfection method is required for an efficient and safe intraperitoneal gene therapy. A transfection method based on BLs with an intravenous route has been established for various tissues, including the liver (Un et al., 2010), kidney (Kurosaki et al., 2014) and tumors (Suzuki et al., 2015). However, the efficiency and safety of transgene expression with an intraperitoneal gene transfection system based on BLs has not been reported. Therefore, we developed an intraperitoneal gene transfection method using BLs with US irradiation. We report here that this US-mediated gene transfection system shows increased transfection efficacy in targeted cells and organs with low cytotoxicity in mice.

We confirmed physicochemical characteristics of BLs. The BLs had nanoscale size and almost neutral surface charge. We checked the stability of BLs with or without US irradiation (Figure S2). It was suggested that BLs were disrupted by US irradiation and the US contrast gas encapsulated in BLs was released within 30 s. In contrast, BLs without US irradiation was enough stable for injection.

We compared BLs with microbubbles, Sonazoid®, for intraperitoneal gene transfection (Figure S3). BLs tended to be increase transgene expression compare with Sonazoid®. This tendency might be due to the differences in particle size and surface charge. It was reported that Sonazoid® had 3 μm size with negative charge (Sontum, 2008). Then, the particle number of Sonazoid® should be lower than that of BLs. Moreover, negative surface charge of Sonazoid® may result in electrostatic repulsion against the cells. Therefore, we considered BLs are suitable for intraperitoneal gene transfection.

The duration of US irradiation, the injection volume, and the dose of BLs influence intraperitoneal transgene expression because these factors affect the cavitation energy (Qiu et al., 2010) and the intraperitoneal pharmacokinetics of macromolecules (Barrett et al., 1991). The optimal conditions are 3 min period of US irradiation, an injection volume of 600 μL, and a dose of BLs of 250 μg (Figure 1). The transgene expression in the peritoneal tissues tended to be related to the duration of US irradiation, whereas the transgene expression was not related to the injection volume or the dose of BLs. Six hundred microliters of solution might be sufficient to fill the peritoneal cavities of mice and to transfer the cavitation energy. Thus, we successfully optimized the transfection conditions for intraperitoneal tissues when BLs and US irradiation are used.

Both BLs and US irradiation play important roles in this highly efficacious transfection. Guo et al. (2007) failed to detect transgene expression in the liver or intestines using microbubbles with US irradiation in rats. In contrast, we detected high transgene expression in these tissues using BLs with US irradiation (Figure 1). However, negligible transgene expression was detected when BLs only or US irradiation only was used, whereas the transgene expression in the peritoneal tissues was enhanced 50–100-fold when BLs were supplemented with US irradiation compared with BLs alone. This enhancement is similar to that reported elsewhere when other tissues were transfected with BLs and irradiation with US (Suzuki et al., 2007). Sonoporation can also achieve selective gene transfection around a US irradiation site. To evaluate the selectivity of intraperitoneal transfection by BLs with US irradiation, we irradiated the liver transdermally with US (Figure 2). Transgene expression increased markedly in the liver and the peritoneal wall, the US-irradiated sites. These data suggest that US irradiation can control the site of transgene expression after the intraperitoneal injection of BLs.

Long-term transgene expression is required to cure chronic diseases. The CpG-depleted pDNA vector is known to prolong the duration of transgene expression because this vector induces a negligible host toll-like receptor 9 (TLR-9)-mediated proinflammatory immune response (Yew et al., 2002). Therefore, to achieve long-term transgene expression, we used pCpGfree–Lucia as the CpG-free pDNA vector. As shown in Figure 3(a), transgene expression in the intraperitoneal fluid was significantly higher than that in serum. This result is consistent with the intraperitoneal transfection achieved with calcium carbonate microflowers (Fumoto et al., 2012). Secretion polarity of the transgene products in epithelial cell lines transfected with cationic liposomes has been reported (Nakanishi et al., 2002), so the secretion polarity of transgene products might be a common phenomenon. The transgene expression in the intraperitoneal fluid lasted for at least 1 month. These results indicate that the intraperitoneal transfection of the CpG-free pDNA vector using BLs with US irradiation can achieve both US-irradiated-site-selective and long-term gene expression.

Transgene expression from pCMV–luciferase in the visceral tissues, such as the liver, using decreased rapidly, whereas the transgene expression in the peritoneal wall persisted for 14 days (Figure 3b). The reason for these different periods of transgene expression is still unclear. We previously observed transgene expression for ca. 1 month after the transfer of pDNA by rubbing gastric surface, whereas its expression did not persist without rubbing (Mine et al., 2011). These findings suggest that the relationship between the physical stimulation of transfection and the target tissue is associated with long-term transgene expression in peritoneal tissues. Further studies are required to elucidate the mechanism underlying long-term transgene expression.

Safety is an obvious concern in intraperitoneal gene transfection systems for therapeutic applications to peritoneal injury. Although microbubbles with US irradiation induce little damage to the peritoneum (Guo et al., 2007), the injury to the peritoneal tissues by sonoporation has not been adequately assessed. Therefore, we determined the safety of this transfection method using BLs with US irradiation in the peritoneal tissues in detail. As shown in Figure 4(a), no morphological changes to the peritoneum of peritoneal wall were observed and the RD-10/FD-2000 ratio, as an indicator of peritoneal function, did not decrease relative to the control ratio (Figure 4b). Moreover, the marker of hepatic toxicity ALT levels in the sera were normal, as in the saline-injected group (Figure 5). These results lead us to conclude that BLs with US irradiation have little effect on the intraperitoneal tissues under the conditions required for efficient gene transfection.

The spatial distribution of transgene expression can provide an important information for gene therapy directed towards peritoneal injuries because the structure of the peritoneal membrane changes during peritoneal injury (Yung & Chan, 2012). We previously assessed spatial distribution of transgene expression by a tissue-clearing method without sectioning (Fumoto et al., 2016). Transgene expression was observed to depths of ca. 100 μm in tissue samples cleared with the SeeDB or Clear^T2^ method. These methods are also compatible with lipophilic carbocyanine dyes, such as DiI. Therefore, we examined transgene expression in a peritoneum surface-labeled with DiI and cleared with the Clear^T2^ method because the thickness of the submesothelial layer is ca. 100 μm in a mouse model of peritoneal fibrosis (Io et al., 2015). Transgene expression in the visceral peritoneum, in the liver and stomach, was only observed on the peritoneal surface cells (Figure 6). In contrast, transgene expression in the peritoneal wall of the parietal peritoneum was observed on the peritoneal surface and the submesothelial layer. These results agreed with the finding of Guo et al., (2007) who observed transgene expression in both mesothelial and submesothelial layers of parietal peritoneum with microbubbles and US irradiation. Therefore, using a tissue-cleaning method, we have demonstrated that BLs with US irradiation can selectively transfect peritoneal cells.

## Conclusions

We have demonstrated the efficiency and safety of intraperitoneal gene transfection using BLs with US irradiation. We also achieved long-term transgene expression in the peritoneal cavity. BLs with US irradiation showed little toxicity against the peritoneal tissues. Moreover, the spatial distribution of the transgene expression in the peritoneal tissues indicated that nontarget parenchymal cells were kept intact in terms of transgene expression. Therefore, BLs with US irradiation may be an effective and safe method of transfection to the peritoneum. This intraperitoneal gene transfection system using BLs with US irradiation will contribute to future gene therapies for peritoneal diseases, including peritoneal fibrosis.

## Supplementary Material

DD-Supp.docx

## Notes

**Supplementary material available online**
