# Supplementary material for: Effective intraperitoneal gene transfection system using nanobubbles and ultrasound irradiation
Source: Drug Deliv. 2017 Apr 27;24(1):737–44. doi: 10.1080/10717544.2017.1319433 (PMC8241157; doi:10.1080/10717544.2017.1319433)
Supplement: DD-Supp.docx [file IDRD_A_1319433_SM7346.docx]

Figure S1. Size distribution of BLs.


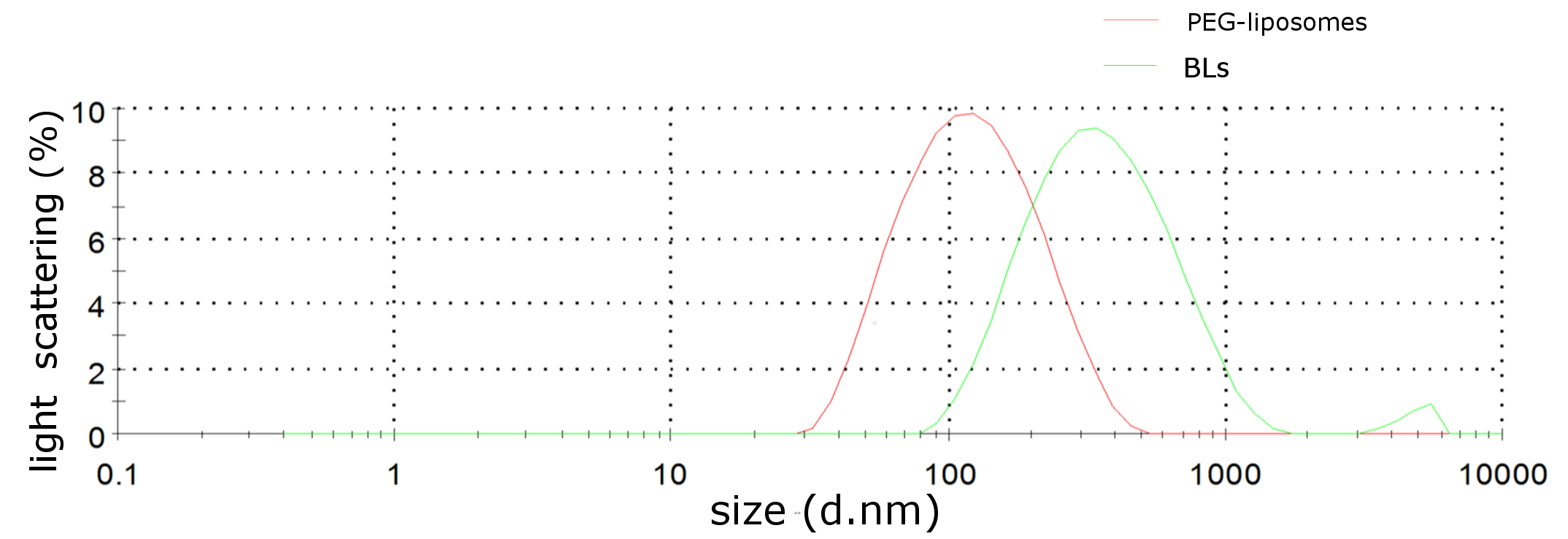


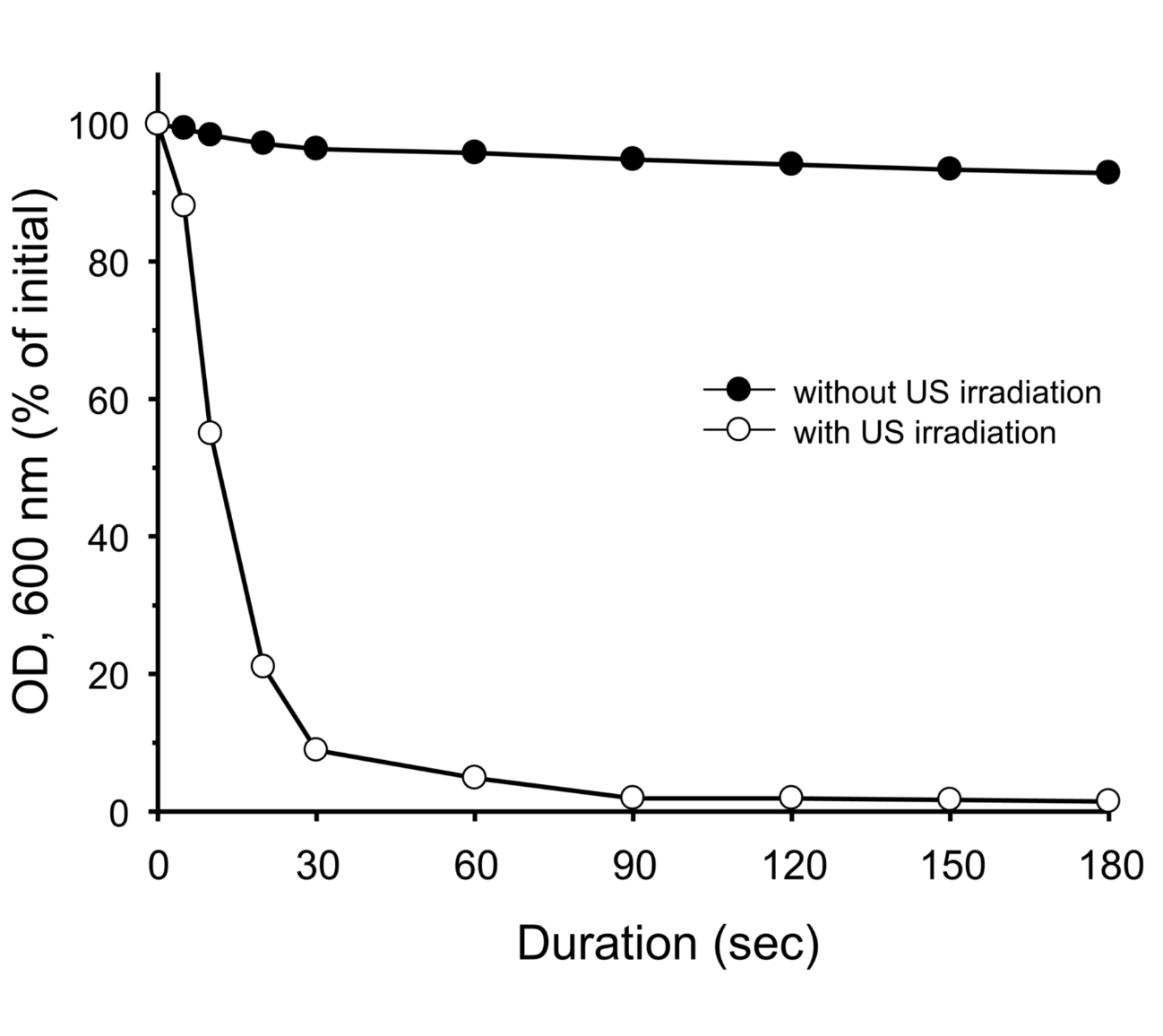
 Figure S2. The stability of BLs with or without US irradiation.

Changes in OD at 600 nm of BLs with (open circle) or without (closed circle) US irradiation were monitored using an ultraviolet and visible spectrophotometer.


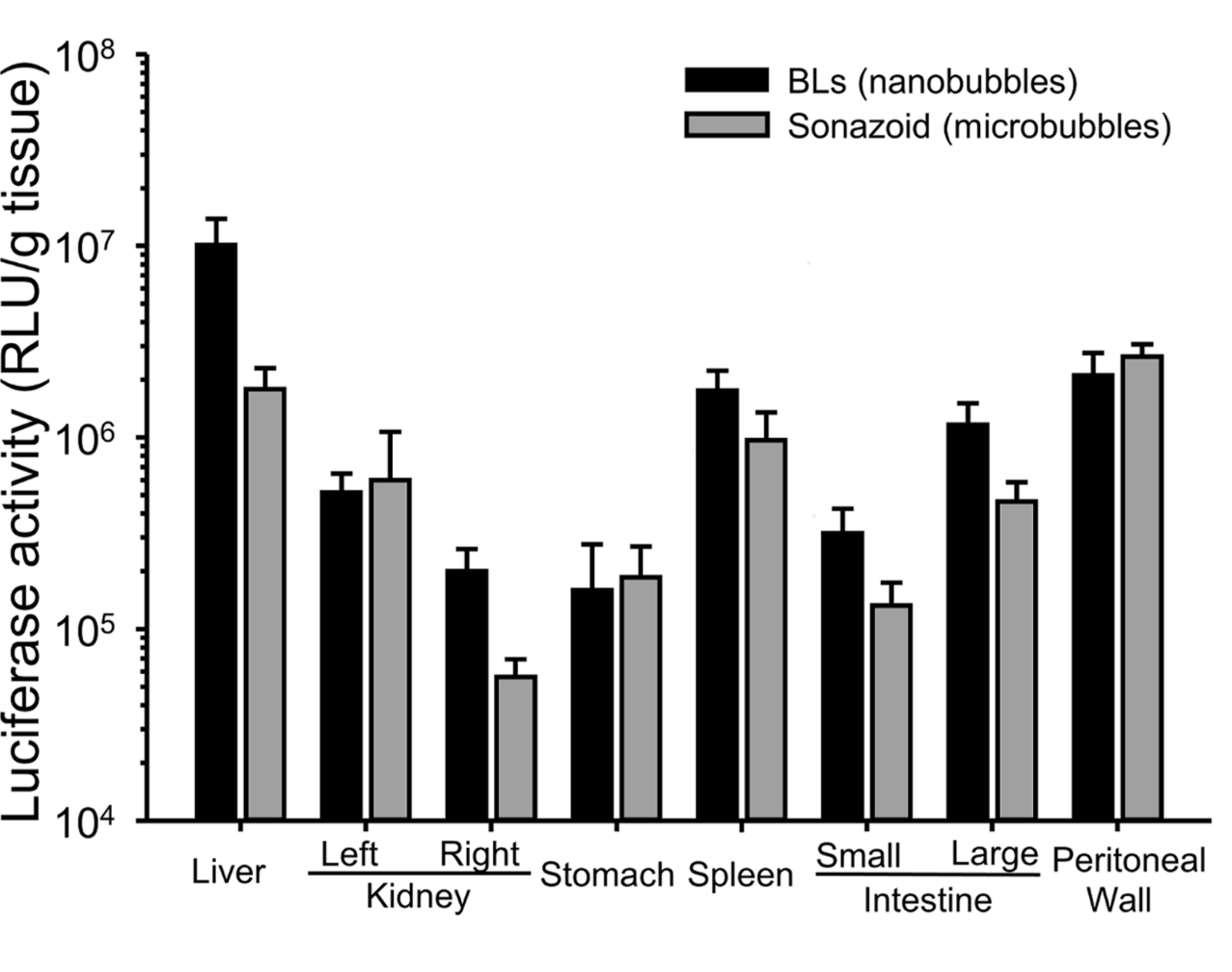
 Figure S3. Comparison of BLs with microbubbles for intraperitoneal gene transfection.

Mice were injected intraperitoneally with a 600 µL mixture of pDNA (60 µg) and BLs (250 µg) or Sonazoid^®^ (250 µg), followed by US irradiation for 2 min. Six hours after injection of pCMV–Luciferase, the luciferase activities were measured. Each bar represents the mean ± SE of four experiments.
